# Lactate dehydrogenase and the severity of adenoviral pneumonia in children: A meta-analysis

**DOI:** 10.3389/fped.2022.1059728

**Published:** 2023-01-26

**Authors:** Min Zou, Yang Zhai, Xiaoping Mei, Xing Wei

**Affiliations:** ^1^ Department of Pediatrics, Guangxi International Zhuang Medicine Hospital, Nanning, China; ^2^ Guangxi Key Laboratory of Chinese Medicine Foundation Research, Guangxi University of Chinese Medicine, Nanning, China; ^3^ International Medical Department, Guangxi International Zhuang Medicine Hospital, Nanning, China; ^4^ Department of Endocrinology, Guangxi International Zhuang Medicine Hospital, Nanning, China; ^5^ Zhuang Medical College, Guangxi University of Chinese Medicine, Nanning, China

**Keywords:** adenoviral pneumonia, lactate dehydrogenase, severity, postinfectious bronchiolitis obliterans, mortality, meta-analysis

## Abstract

**Background:**

Children with severe adenoviral pneumonia (ADVP) have poor prognosis and high risk of mortality. We performed a meta-analysis to evaluate the association between pretreatment lactate dehydrogenase (LDH) and severity, postinfectious bronchiolitis obliterans (PIBO), and mortality in children with ADVP.

**Methods:**

Relevant observational studies were identified by search of PubMed, Embase, Web of Science, Wanfang, and CNKI databases from inception to August 3, 2022. A random effect model was used to pool the results by incorporating the potential between-study heterogeneity.

**Results:**

Overall, 23 studies with 4,481 children with ADVP were included in this meta-analysis. Results of meta-analysis showed that children with severe ADVP had a significantly higher level of pretreatment LDH as compared to those with non-severe ADVP (standard mean difference [SMD]: 0.51, 95% confidence interval [CI]: 0.36 to 0.66, *p* < 0.001; *I*^2^ = 69%). Besides, pooled results also suggested that the pretreatment LDH was significantly higher in children who developed PIBO as compared to those who did not (SMD: 0.47, 95% CI: 0.09 to 0.84, *p* = 0.02, *I*^2^ = 80%). Finally, results of the meta-analysis also confirmed that a higher pretreatment LDH (>500 IU/L) was a risk factor of increased mortality during hospitalization (odds ratio: 3.10, 95% CI: 1.62 to 5.92, *p* < 0.001, *I*^2^ = 0%). Sensitivity analyses by excluding one dataset at a time showed consistent results.

**Conclusion:**

High pretreatment LDH may be associated with disease severity, development of PIBO, and increased risk of mortality in children with ADVP.

## Introduction

Adenovirus belongs to the family Adenoviridae, which is characterized by its non-enveloped and double-stranded DNA properties (1). It has been discovered that there are at least 90 genotypes of human adenovirus, which can be divided into seven species from A to G (2). As a result of adenovirus infection, a variety of illnesses can be acquired, including bronchitis, pneumonia, conjunctivitis, gastroenteritis, and hemorrhagic cystitis (3). In children, adenoviral pneumonia (ADVP) has become an important cause of mortality, particularly for those <5 years and suffering from severe pneumonia (4, 5). According to a statistic report from some southern cities in China in 2018–2019, ADVP accounts for approximately 25% of the overall community acquired pneumonia in children (6). In addition, the early mortality of children with ADVP was reported as high as 50% (7). For children who survived from ADVP, severe complication may develop, such as the postinfectious bronchiolitis obliterans (PIBO) (8). In general, PIBO is the predominant type of bronchiolitis obliterans in children which is characterized of persistent airway obstruction with functional and radiological evidence of small airway involvement after respiratory infection (9). The clinical consequences of PIBO include impaired pulmonary function and even respiratory failure in later life (9). However, clinical parameters that are closely related to the disease severity, development of PIBO and early mortality in children with ADVP remain to be determined (10).

The lactate dehydrogenase (LDH) enzyme plays a vital role in the anaerobic metabolism of the body (11, 12). It is ubiquitously present in all cells and responds to tissue damage in a nonspecific manner. For coronavirus disease 2019 (COVID-19), accumulating evidence suggests that the level of LDH could be used as a maker of disease severity (13). As a result of the reemergence of the adenovirus epidemic in central and southern China in 2018 and 2019, many studies have been conducted to evaluate the prognosis factors associated with ADVP among children, including the clinical significance of serum LDH (14–36). In this meta-analysis, we systematically evaluated the relationships of LDH at baseline with disease severity, risk of PIBO, and in-hospital mortality of children with ADVP. The findings are expected to provide information regarding early risk stratification in children with ADVP.

## Materials and methods

The Preferred Reporting Items for Systematic Reviews and Meta-Analyses (PRISMA) statement (37, 38) and the Cochrane's Handbook (39) guideline was followed in the conceiving, conducting, and reporting the study.

### Search of databases

Studies were retrieved by search electronic databases including PubMed, Embase, Web of Science, Wanfang, and CNKI (China National Knowledge Infrastructure) databases from inception to August 3, 2022, with combined search terms including (1) “lactate dehydrogenase” OR “LDH”; (2) “adenovirus” OR “adnoviral” OR “ADV”; and (3) “pneumonia” OR “respiratory” OR “infection”. The search was restricted to human studies with no limitation of the publication language. The reference lists of the relevant original and review articles were also manually screened for possible related studies.

### Study inclusion and exclusion criteria

We formulated the inclusion criteria according to the aim of the meta-analysis, with the following specified inclusion criteria: (1) designed as observational studies, including the case-control study, cross-sectional study, and cohort study; (2) included children with confirmed diagnosis of ADVP; (3) serum level of LDH was measure at baseline (within 24 h of admission); and (4) reported the association between LDH with at least one of the following outcomes, including the severity of ADVP, the development of PIBO, and the all-cause mortality during hospitalization. We did not apply restriction for the definition of disease severity of ADVP during the selection of the studies. This is mostly because there has been no international consensus regarding the classification according to the severity of ADV-pneumonia. However, because all of the retrieved studies were performed in China and the severity of ADVP was evaluated according to the Chinese guidelines, we therefore used these criteria in our meta-analysis accordingly. Specifically, a severe ADVP was diagnosed based on the criteria of the 2019 Chinese Guideline for the Diagnosing and Management of Children with Community-Acquired Pneumonia (40) if any of the following criteria were met: (1) Overall poor health condition of a patient. (2) A conscious disturbance. (3) Cyanosis or tachypnea [age <2 months: respiratory rate (RR) 60 breaths/minute; age 2 months to 1 year: RR 50 breaths/minute; age 1–5 years: RR 40 breaths/minute; and age >5 years: RR 30 breaths/minute)], intermittent apnea, or oxygen saturation of <92%. (4) Persistent hyperpyrexia or ultrahyperpyrexia for more than 5 days. (5) Dehydration or food refusal. (6) A chest x-ray or CT reveals pulmonary infiltration of at least two-thirds of the lung, pneumothorax, lung necrosis, or lung abscess on one side. (7) Extrapulmonary complications. Diagnosis of PIBO was consistent with the criteria applied in previous studies (41): (1) continuous or repeated coughing and wheezing for 6 weeks after the infection; (2) evidence of obstructive pulmonary disease on computed tomography (CT) examination imaging such as hyperventilation, atelectasis, bronchial wall thickening, bronchiectasis and mosaic perfusion, and air trapping (two fixed radiologists performed imaging reports); (3) excluding other chronic pulmonary diseases such as tuberculosis, cystic fibrosis, bronchopulmonary congenital dysplasia, and primary immune deficiency. Only studies published as full-length articles were included. Reviews, studies not including children with ADVP, studies not evaluating pretreatment LDH, or studies not reporting the outcome of interest were excluded.

### Data collection and quality assessing

The literature search, data collection, and study quality assessment were independently conducted by two authors separately. If discrepancies occurred, a third author was contacted for discussion and reaching the consensus. We collected data regarding study information, design, diagnosis of the children, age, timing of LDH measurement, follow-up durations, and outcomes reported. Study quality was assessed *via* the Newcastle–Ottawa Scale (42) with scoring regarding the criteria for participant selection, comparability of the groups, and the validity of the outcomes. The scale ranged between 1 and 9 stars, with larger number of stars presenting higher study quality.

### Statistical analyses

The differences of LDH levels admission between children with severe and non-severe ADVP, and between those who developed and did not develop PIBO were summarized as standard mean difference (SMD) and the corresponding 95% confidence interval (CI). The association between high LDH at baseline and the risk of all-cause mortality during hospitalization in children with ADVP was summarized as odds ratio (OR) and 95% CI. Using the 95% CIs or *p* values, data of OR and the standard error (SE) could be calculated, and a subsequent logarithmical transformation was conducted to keep stabilized variance and normalized distribution. Between study heterogeneity was estimated with the Cochrane's Q test and the *I*^2^ statistic (43), with *I*^2^ > 50% reflecting the significant heterogeneity. A random-effect model was applied to combine the results by incorporating the influence of heterogeneity (39). Sensitivity analysis by excluding one dataset at a time was performed to evaluate the influence of individual study on the meta-analysis results (44). For meta-analyses with adequate datasets (at least ten) the publication bias was estimated based on the visual judgement of the symmetry of the plots, supplemented with the Egger's regression asymmetry test (45). The RevMan (Version 5.1; Cochrane Collaboration, Oxford, United Kingdom) and Stata software (version 12.0; Stata Corporation, College Station, TX) were applied for these analyses.

## Results

### Literature search

**Figure 1:**
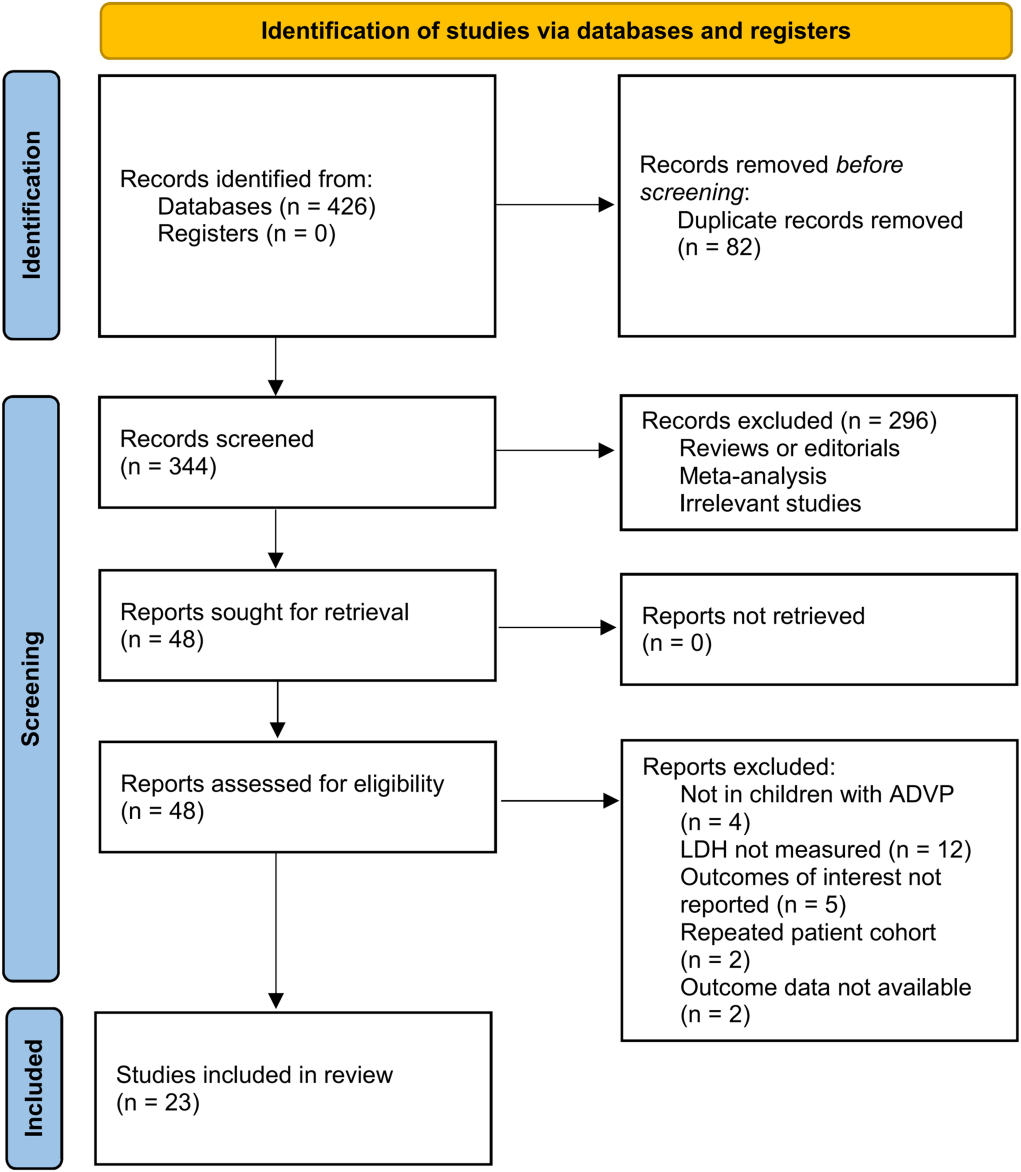
Diagram of database search and study inclusion.

The flowchart of literature search and study inclusion was displayed in Figure 1. In summary, 426 records were obtained in the initial database search, and 82 duplications were removed. Subsequently, 296 studies were further removed after screening with titles and abstracts, largely because they were not relevant to the objective of the meta-analysis. Finally, 48 studies underwent full-text review, and 25 of them were excluded for the reasons listed in Figure 1. Accordingly, 23 studies were available for the meta-analysis (14–36).

### Study characteristics

Overall, 23 studies (14–36) involving 4,481 children with confirmed diagnosis of ADVP were included in the meta-analysis. Generally, children with confirmed diagnosis of ADVP without known immunodeficiency or history of receiving immunosuppressive agents were included in these studies. The characteristics of the included studies are displayed in Table 1. Briefly, these studies were all retrospective cohort studies from China, and published between 2020 and 2022. The serum level of LDH was measured within 24 h of admission for all the included patients. For all the included studies, clinically diagnosed pneumonia with nasopharyngeal swab positive for adenovirus nucleic acid, serum adenovirus-specific IgM antibody positive, or detected adenovirus nucleic acid sequences in bronchoalveolar lavage fluid (BALF) and metagenomics next generation sequencing (mNGS) in severity cases were all considered as the confirmation of the diagnosis of ADVP. The treatments during hospitalization were only reported in eight studies (15, 18, 20, 22, 23, 34–36), which included symptomatic and supporting treatments, oral prednisone and intravenous steroids, intravenous immunoglobulin, antiviral ribavirin, and respiratory support if necessary (Table 1). The follow-up durations were within hospitalization in most of the included studies, while others were with a follow-up duration of 1 month (36), 3 months (20, 35), and 12 months (18, 23, 32). As for the outcomes reported, 14 studies reported the difference of LDH level at baseline between children with severe and non-severe ADVP (14, 16, 17, 19, 21, 24–31, 33), 6 studies reported the difference of LDH level at baseline between children who developed and did not develop PIBO (18, 20, 23, 32, 35, 36), and three studies reported the association between LDH admission and in-hospital mortality of children with ADVP (15, 22, 34). The NOS of the included studies were all six to seven stars, suggesting moderate to good study quality (Table 2).

**Table 1:** Characteristics of the included studies.

Study	Country	Design	Patients	Sample size	Age	LDH measurement timing	Treatment	Follow-up durations	Outcomes reported
Xu 2020	China	RC	Children with ADVP	285	1 to 5 years, mean: 1.7 years	Within 24 h of admission	NR	During hospitalization	Severe vs. non-severe
Zeng 2020	China	RC	Children with ADVP	151	1 month to 14 years	Within 24 h of admission	Symptomatic and supporting treatments, and IVIG if necessary	During hospitalization	OR for mortality (LDH >500)
Shen 2021	China	RC	Children with ADVP	122	1 to 5 years, mean: 3 years	Within 24 h of admission	NR	During hospitalization	Severe vs. non-severe
Zhou 2021	China	RC	Children with ADVP	159	Mean: 2.1 years	Within 24 h of admission	NR	During hospitalization	Severe vs. non-severe
Zhong 2021	China	RC	Children with ADVP	211	3 months to 8 years	Within 24 h of admission	NR	During hospitalization	Severe vs. non-severe
Lou 2021	China	RC	Children with ADVP	168	5 months to 9 years, median: 2.6 years	Within 24 h of admission	NR	During hospitalization	Severe vs. non-severe
Huang 2021a	China	RC	Children with ADVP	85	5 months to 6 years, median: 1.3 years	Within 24 h of admission	Symptomatic and supporting treatments, oral prednisone and respiratory support for children with PIBO	12 months	PIBO vs. no PIBO
Huang 2021b	China	RC	Children with ADVP	204	5 months to 6 years, median: 2.1 years	Within 24 h of admission	NR	During hospitalization	Severe vs. non-severe
Huang 2021c	China	RC	Children with severe ADVP	45	Median 1.7 years	Within 24 h of admission	Symptomatic and supporting treatments, and respiratory support for children with PIBO	3 months	PIBO vs. no PIBO
Huang 2021d	China	RC	Children with ADVP	60	1 to 15 years, mean: 8.4 years	Within 24 h of admission	NR	During hospitalization	Severe vs. non-severe
Li 2021	China	RC	Children with ADVP	102	Mean: 3.1 years	Within 24 h of admission	Symptomatic and supporting treatments, oral prednisone and respiratory support for children with PIBO	12 months	PIBO vs. no PIBO
Liu 2021	China	RC	Children with ADVP	131	NR	Within 24 h of admission	NR	During hospitalization	Severe vs. non-severe
Gu 2021	China	RC	Children with ADVP	467	Median: 1.9 years	Within 24 h of admission	NR	During hospitalization	Severe vs. non-severe
Huang 2021e	China	RC	Children with severe ADVP	75	3 months to 8 years, median: 2.3 years	Within 24 h of admission	Symptomatic and supporting treatments, antiviral ribavirin, and IVIG if necessary	During hospitalization	OR for mortality (LDH >500)
Hu 2021	China	RC	Children with ADVP	541	1 month to 16 years, median: 2.6 years	Within 24 h of admission	NR	During hospitalization	Severe vs. non-severe
Feng 2022	China	RC	Children with ADVP	488	NR	Within 24 h of admission	NR	During hospitalization	Severe vs. non-severe
Peng 2022	China	RC	Children with ADVP	453	0-14 years, mean: 2.4 years	Within 24 h of admission	NR	12 months	PIBO vs. no PIBO
He 2022	China	RC	Children with ADVP	177	Mean: 2.4 years	Within 24 h of admission	NR	During hospitalization	Severe vs. non-severe
Wang 2022a	China	RC	Children with severe ADVP	303	Median: 1.3 years	Within 24 h of admission	Symptomatic and supporting treatments, respiratory support, and IVIG if necessary	During hospitalization	OR for mortality (LDH >500)
Tang 2022	China	RC	Children with ADVP	84	Mean: 3.4 years	Within 24 h of admission	NR	During hospitalization	Severe vs. non-severe
Wen 2022	China	RC	Children with ADVP	60	0-5 years, mean: 1.1 years	Within 24 h of admission	Symptomatic and supporting treatments, and IVIG if necessary	1 months after discharge	PIBO vs. no PIBO
Li 2022	China	RC	Children with ADVP	32	Mean: 3.8 years	Within 24 h of admission	NR	During hospitalization	Severe vs. non-severe
Wang 2022b	China	RC	Children with severe ADVP	78	Mean: 1.1 years	Within 24 h of admission	Symptomatic and supporting treatments, respiratory support, and IVIG if necessary	3 months	PIBO vs. no PIBO

RC, retrospective cohort; ADVP, adenoviral pneumonia; LDH, lactate dehydrogenase; OR, odds ratio; PIBO, postinfectious bronchiolitis obliterans; IVIG, intravenous immunoglobulin.

**Table 2:** Study quality evaluation *via* the Newcastle-Ottawa scale.

Study	Representativeness of the exposed cohort	Selection of the non-exposed cohort	Ascertainment of exposure	Outcome not present at baseline	Control for age	Control for other confounding factors	Assessment of outcome	Enough long follow-up duration	Adequacy of follow-up of cohorts	Total
Xu 2020	1	1	1	1	0	0	1	1	1	7
Zeng 2020	1	1	1	1	0	0	1	1	1	7
Shen 2021	1	1	1	1	0	0	1	1	1	7
Zhou 2021	1	1	1	1	0	0	1	1	1	7
Zhong 2021	1	1	1	1	0	0	1	1	1	7
Lou 2021	1	1	1	1	0	0	1	1	1	7
Huang 2021a	1	1	1	1	0	0	1	1	1	7
Huang 2021b	1	1	1	1	0	0	1	1	1	7
Huang 2021c	0	1	1	1	0	0	1	1	1	6
Huang 2021d	1	1	1	1	0	0	1	1	1	7
Li 2021	1	1	1	1	0	0	1	1	1	7
Liu 2021	1	1	1	1	0	0	1	1	1	7
Gu 2021	1	1	1	1	0	0	1	1	1	7
Huang 2021e	0	1	1	1	0	0	1	1	1	6
Hu 2021	1	1	1	1	0	0	1	1	1	7
Feng 2022	1	1	1	1	0	0	1	1	1	7
Peng 2022	1	1	1	1	0	0	1	1	1	7
He 2022	1	1	1	1	0	0	1	1	1	7
Wang 2022a	0	1	1	1	0	0	1	1	1	6
Tang 2022	1	1	1	1	0	0	1	1	1	7
Wen 2022	1	1	1	1	0	0	1	1	1	7
Li 2022	1	1	1	1	0	0	1	1	1	7
Wang 2022b	0	1	1	1	0	0	1	1	1	6

### Meta-analysis results

**Figure 2:**
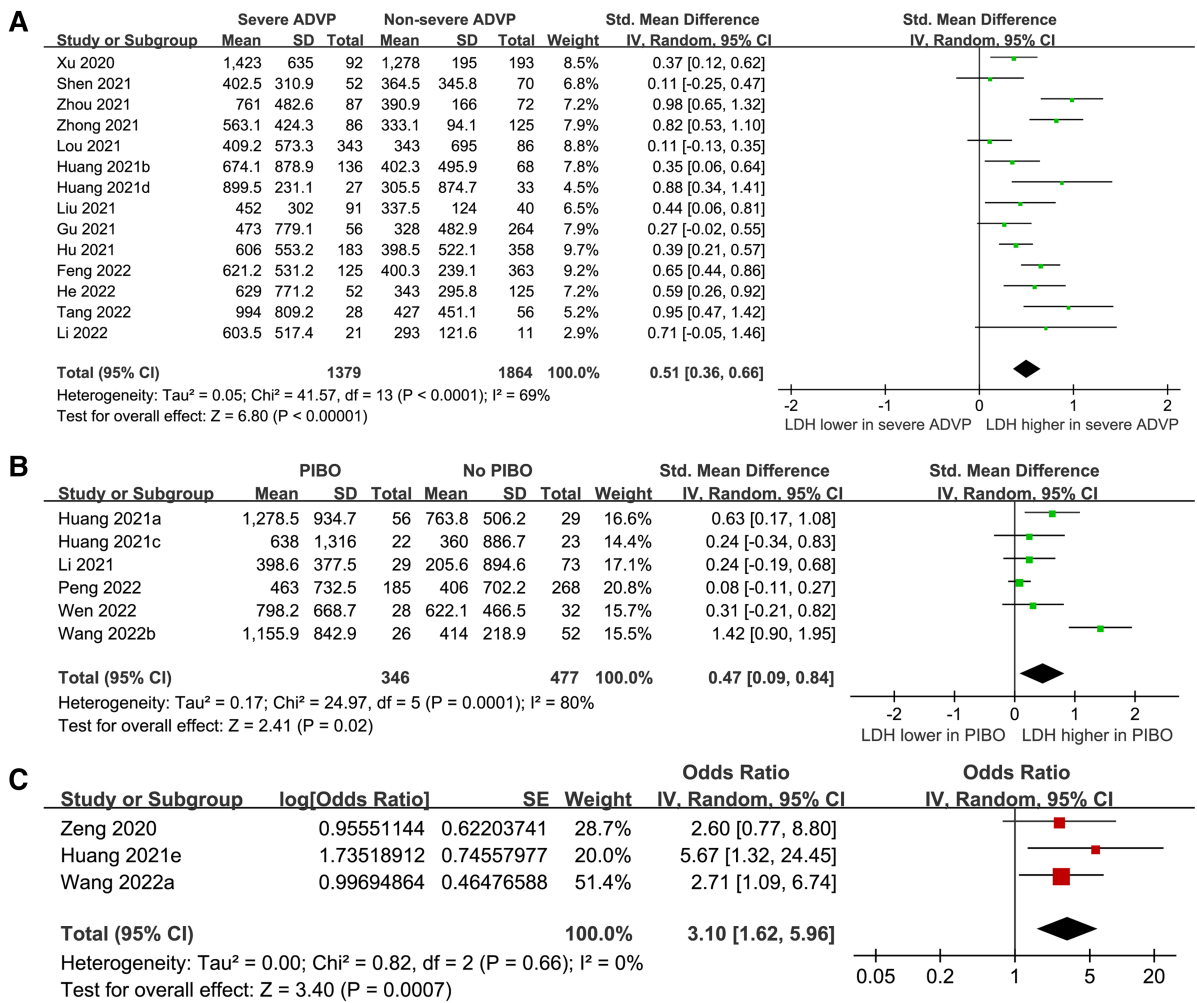
Forest plots for the meta-analyses of the role of LDH in children with ADVP. (**A**), meta-analysis for the difference of serum LDH between children with severe and non-severe ADVP; (**B**), meta-analysis for the difference of serum LDH between children who developed and did not develop PIBO; and (**C**), meta-analysis for the association between high LDH (> 500 IU/L) and in-hospital mortality risk in children with ADVP.

Pooled results of 14 studies (14, 16, 17, 19, 21, 24–31, 33) with a random-effect model showed that children with severe ADVP had a significantly higher level of pretreatment LDH as compared to those with non-severe ADVP (SMD: 0.51, 95% CI: 0.36 to 0.66, *p* < 0.001; *I*^2^ = 69%; Figure 2A). Sensitivity analyses by excluding one study at a time showed similar results (SMD: 0.47 to 0.54, *p* all < 0.05).

Besides, pooled results of 6 studies (18, 20, 23, 32, 35, 36) indicated that the pretreatment LDH was significantly higher in children who developed PIBO as compared to those who did not (SMD: 0.47, 95% CI: 0.09 to 0.84, *p* = 0.02, *I*^2^ = 80%; Figure 2B). Sensitivity analysis by omitting one study at a time also showed consistent results (SMD: 0.23 to 0.57, *p* all < 0.05).

Finally, pooling the results of three studies (15, 22, 34) showed that a higher pretreatment LDH (>500 IU/L) was a risk factor of increased mortality during hospitalization (OR: 3.10, 95% CI: 1.62 to 5.92, *p* < 0.001, *I*^2^ = 0%; Figure 2C). Sensitivity analyses by excluding one study at a time showed consistent results (OR: 2.67 to 3.58, *p* all < 0.05).

### Publication bias

**Figure 3:**
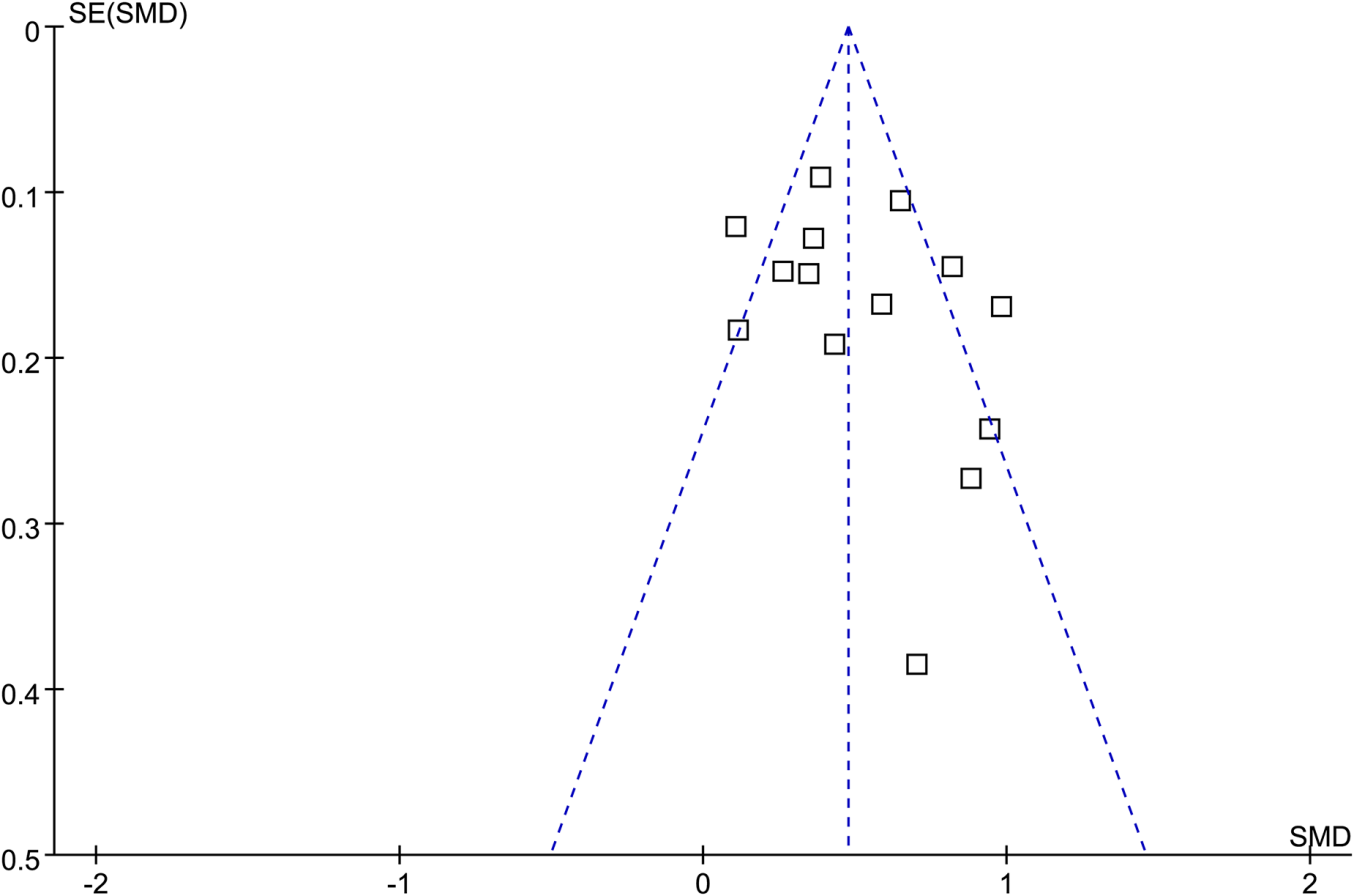
Funnel plots for the publication bias underlying the meta-analysis for the difference of serum LDH between children with severe and non-severe ADVP.

Figure 3 displays the funnel plots for the meta-analysis of the difference of the LDH between children with severe and non-severe ADVP. Visual inspection revealed symmetry of the plots, reflecting a low risk of publication biases. The Egger's regression tests also indicated low risk of publication biases (*p* = 0.31). The publication biases for the meta-analyses of the other two outcomes were difficult to estimate because less than 10 studies were included.

## Discussion

In this study, by pooling the results of eligible observational studies, results of the meta-analysis showed that children with severe ADVP had a significantly higher level of baseline LDH as compared to those with non-severe ADVP. Besides, the serum level of LDH was also higher in children who developed PIBO as compared to those who did not develop PIBO within 12 months. Finally, a high pretreatment LDH > 500 IU/L may be a predictor of early mortality of children with ADVP during hospitalization. Collectively, results of the meta-analysis suggest that for children with ADVP, a higher LDH level at admission is associated with the severity of the disease, risk for the development of PIBO, and the all-cause mortality during hospitalization.

To the best of our knowledge, this is the first meta-analysis regarding the role of LDH in the disease severity evaluation and prognostic prediction in children with ADVP. The strengths of the meta-analysis include extensive literature search to retrieve eligible studies from both the English and Chinses databases, comprehensive evaluation of three outcomes such as disease severity, development of PIBO, and risk of all-cause mortality, as well as the performance of sensitivity analyses to indicate the robustness of the findings. Previous studies have shown that several lung conditions affect serum LDH levels (46), and patients with severe adenovirus respiratory tract infections have elevated LDH levels (47). In addition, a recent study showed that the LDH level was the associated factor to predict the types of pneumonia, which was significantly higher in children with ADVP as compared to those with bacterial pneumonia (48). In this meta-analysis, we further confirmed that higher baseline LDH may be a marker of disease severity and predictor of PIBO and in-hospital mortality in children with ADVP, which further supported the role of LDH as an important marker for the diagnosis and clinical management of ADVP. From the clinical perspective, serum LDH could be easily measured at admission in the routine biochemical blood test for these children, which reinforced the potential clinical significance of LDH for children with ADVP.

The mechanisms underlying the association between high LDH and the disease severity and poor prognosis of children with ADVP remain not fully determined. Almost all organ system cells contain the enzyme LDH, which catalyzes the conversion of pyruvate and lactate, with simultaneous conversion of NADH and NAD + (49). Five separate isozymes exist in humans, including LDH-1 in cardiomyocytes, LDH-2 in reticuloendothelial system, LDH-3 in pneumocytes, LDH-4 in kidneys and pancreas, and LDH-5 in liver and striated muscle (49). Besides of its role as a traditional marker of cardiac injury, infections and tissue trauma could also lead to increased LDH, that result in increased lactate trigger metalloprotease activation and increase macrophage-mediated angiogenesis (50). Considering that lung tissue (isozyme 3) contains LDH, children with severe ADVP can be expected to have higher levels of LDH in their bloodstreams. However, there is no evidence that the different LDH isoenzymes contribute to the LDH elevation observed in severe ADVP. Besides, accumulating evidence suggests that elevated LDH may be a maker of over-activated inflammation of pulmonary tissue (46) and compromised immune response, which are both associated with poor prognosis of patients with severe infection (12). Studies are needed in the future to determine the accurate mechanisms underlying the relationships of high LDH with disease severity and poor prognosis of ADVP.

Our study has limitations. First, all of the studies included in the meta-analysis were performed in China, which may primarily due to the reemergence of epidemic of the adenovirus infection in China in 2018–2019. Therefore, studies are needed to validate the potential role of LDH in ADVP in children from other countries. Besides, the severity of ADVP was evaluated according to the Chinese guidelines in this study. Correlations of LDH with more practical parameters for evaluating the severity of ADVP should be investigated in the future, such as the odds of mechanical ventilations, incidence of multi-organ failure, and the proportions of patients requiring extracorporeal membrane oxygenation etc. Second, significant heterogeneity was observed for the meta-analyses of the differences of LDH between severe and non-severe ADVP, and between children who developed and did not develop PIBO. These heterogeneities may be explained by the potential differences of adenoviral type, antiviral treatments, and related comorbidities etc. among the included studies. However, we were unable to determine the exact source of heterogeneity because the above characteristics mentioned were rarely reported in the original studies. Moreover, comorbidities potentially affecting serum LDH level may confound the results. Similarly, co-infection of the pathogens which may affect LDH may also confound the results, such as the co-infections of Pneumocystis jirovecii (51). In addition, all the included studies were retrospective, which were associated with the risks of recall and selection biases. Therefore, large-scale prospective studies are needed to validate our findings. Finally, the normal range for total LDH is 160 to 450 IU/L in newborns, 60 to 170 IU/L in children, and 140 to 280 units/L IU/L in adults (52). However, the optimal cutoff of LDH at baseline to predict the clinical severity and poor prognosis in children with ADVP remains unknown. Studies are warranted in the future in this regard.

To sum up, results of the meta-analysis indicate that for children with ADVP, a higher LDH level at admission was associated with the severity of the disease, risk for the development of PIBO, and the all-cause mortality during hospitalization. These findings support that serum LDH may be applied as potential marker of disease severity and poor prognosis for children with ADVP. Studies may be considered to evaluate whether measuring LDH for predicting disease severity could optimize the treatments for children with severer ADVP.

## Data Availability

The original contributions presented in the study are included in the article/Supplementary Material, further inquiries can be directed to the corresponding author.
